# Impact of point-of-care C-reactive protein testing intervention on non-prescription dispensing of antibiotics for respiratory tract infections in private community pharmacies in Nigeria: a cluster randomized controlled trial

**DOI:** 10.1016/j.ijid.2022.12.006

**Published:** 2023-02

**Authors:** Augustine Onwunduba, Obinna Ekwunife, Ebuka Onyilogwu

**Affiliations:** 1 Department of Pharmaceutical Microbiology and Biotechnology, Nnamdi Azikiwe University, Awka, Nigeria; 2 Department of Clinical Pharmacy and Pharmacy Management, Nnamdi Azikiwe University, Awka, Nigeria; 3 Department of Pure and Industrial Chemistry, Nnamdi Azikiwe University, Awka, Nigeria

**Keywords:** Antibiotics, C-reactive protein, Community pharmacies, Cluster randomized controlled trial, Respiratory tract infections

## Abstract

**Objectives:**

To ascertain if access to C-reactive protein (CRP) test kits—and staff training on how to use them in respiratory tract infection (RTI) management—in private community pharmacies (PCPs) can reduce non-prescription antibiotic dispensing for RTI.

**Methods:**

A parallel cluster randomized controlled trial was conducted in Nigeria. The clusters—which were equally the participating units—were PCPs with blood testing experience. Stratified block randomization was done. PCPs were stratified by the baseline value of the primary outcome. PCPs were not blinded. The intervention PCPs were provided with CRP kits and trained to use them to make decisions regarding non-prescription antibiotic dispensing for RTI. The control PCPs received no intervention. The primary outcome was the non-prescription antibiotic dispensing rate for RTI. Data were collected by blinded simulated clients who visited each PCP 30 times before and after the intervention without prescriptions. Analyses were by intention-to-treat.

**Results:**

Twenty PCPs were randomized, 1:1. Ten PCPs were analyzed in each arm. Each PCP contributed 30 data points to the multiple imputation analysis where antibiotic dispensing decreased by 15.66% (209/300 [intervention] vs 256/300 [control]) in the adjusted analysis (odds ratio = 0·279, 95% confidence interval = 0.107-0.726; *P*-value = 0.0090) and 16% (208/300 [intervention] vs 256/300 [control]) in the crude analysis (odds ratio = 0.299, 95% confidence interval = 0.098-0.911; *P*-value = 0.034).

**Conclusion:**

Access to CRP kits—and staff training on how to use them in RTI management—in PCPs reduced non-prescription antibiotic dispensing for RTI.

## Highlights

• Most respiratory tract infections (RTI) do not require antibiotics.
• In Nigerian private community pharmacies, non-prescription use of antibiotics for RTI is usual.
• Such practice could contribute to the spread of antibiotic resistance.
• C-reactive protein testing intervention in these pharmacies reduces their antibiotic use for RTI.

## Introduction

Many bacteria in Nigeria have developed resistance to antibiotics [1]. This results in severe illnesses from infections and higher healthcare costs. The inappropriate use of antibiotics is one of the factors that facilitate the spread of resistant bacteria [2,3].

Respiratory tract infection (RTI) is caused by viruses and bacteria, however, a majority of cases are caused by viruses [4]. It is often difficult to immediately distinguish viral and bacterial RTI in the absence of specialized diagnostic tools [5]. Consequently, clinicians, in the absence of such tools, are usually tempted to recommend antibiotics to patients with RTI in an attempt to not miss a potentially serious bacterial RTI [6]. This practice is problematic, considering that patients with viral RTI will not benefit from antibiotics, and such unnecessary use of antibiotics facilitates the spread of antibiotic resistance [3].

According to the Federal House of Representatives of Nigeria, there is a shortage of primary healthcare centers in Nigeria, with less than 20% of the 30,000 primary healthcare centers in Nigeria functioning [7]. Consequently, private community pharmacies (PCPs)—rather than primary healthcare centers—routinely provide primary healthcare services [8]. In Nigeria, staff of PCPs includes pharmacists and pharmacy assistants. The pharmacists have Bachelor of Pharmacy and/or Doctor of Pharmacy degrees. They manage PCPs. Pharmacy assistants are well-trained by pharmacists to assist in the day-to-day running of PCPs. They are supervised by pharmacists. PCPs provide consultation services to patients and then dispense medications accordingly or refer them to hospitals [8]. This role played by PCPs is very important as it ensures that primary healthcare is not completely absent in many communities in Nigeria. However, it has its demerits, one of the most important is that it could encourage the unnecessary use of antibiotics, as antibiotics are frequently dispensed to patients without prescriptions.

Accordingly, most patients in Nigeria with RTI receive their treatments from PCPs [8]. In their study to assess the non-prescription dispensation of antibiotics to patients with RTI by PCPs in Nigeria, Akpan et al. [9] found that antibiotics were recommended to 68% of such patients.

Plasma C-reactive protein (CRP) rises well above normal when there is a bacterial infection [10], and thus plasma CRP level can be used to identify bacterial RTI. CRP testing is effective at reducing the unnecessary use of antibiotics for RTI without exposing patients to risks [11].

This study aimed to ascertain if providing PCPs in Nigeria with CRP test kits and training them on how to use them accordingly in the management of RTI has the potential to improve their objective assessment of antibiotic needs in patients with RTI without prescriptions and reduce their dispensation of antibiotics to such patients. Table 1 shows the research in context.

**Table 1:** Research in context.

	Details
Evidence before this study	We searched the title, abstract, and keywords in Scopus using the following terms: ( (respiratory AND tract AND infection*) OR ( upper AND respiratory AND tract AND infection* ) OR rti OR urti ) AND ( antibiotic* ) AND ( ( c-reactive AND protein ) OR ( c AND reactive AND protein ) OR crp ) AND ( pharmacy OR pharmacies ). No restrictions were placed on the search. Two studies that assessed the impact of access to CRP test kits—and staff training on how to use them in RTI management—in PCPs on the rate at which patients with RTI subsequently visit general practitioners with the hope of getting antibiotic prescriptions were found. They were conducted in the United Kingdom and Australia.
The added value of this study	The studies found through our search did not assess the impact of access to CRP test kits—and staff training on how to use them in RTI management—on the rate of non-prescription dispensing of antibiotics for RTI. Instead, they assessed the impact of access to CRP test kits—and staff training on how to use them in RTI management—on the rate at which patients with RTI subsequently visit general practitioners with the hope of getting antibiotic prescriptions. This means that no study has assessed the impact of access to CRP test kits—and staff training on how to use them in RTI management—on the rate of non-prescription dispensing of antibiotics for RTI in PCPs, a situation that is frequently seen in resource-limited settings. This study addresses this gap by assessing how access to CRP test kits—and staff training on how to use them in RTI management—impacts the rate at which PCPs objectively assess antibiotic needs in patients with RTI without prescriptions and the rate at which they dispense antibiotics to such patients.
Implications of all the available evidence	Access to CRP test kits—and staff training on how to use them in RTI management—in PCPs in Nigeria improved the objective assessment of antibiotic needs in patients with RTI without prescriptions and reduced the dispensation of antibiotics to such patients. All things being equal, if economic analysis shows that the cost of implementing this intervention in resource-limited settings is manageable, its implementation by relevant stakeholders in such settings is recommended, as this would help reduce the burden of antibiotic resistance.

CRP, C-reactive protein; PCPs, private community pharmacies; RTI, respiratory tract infection.

## Methodology

### Study design

This trial was a parallel cluster randomized controlled trial. The clusters were PCPs. Cluster randomization was used because clusters were the targets of the intervention. The trial was conducted in Awka, Nigeria. There are over 60 PCPs in Awka. The trial started on January 21, 2022, and ended on July 23, 2022.

### Participants

The trial clusters, PCPs, were also the participating units in this trial. A PCP was considered eligible to be included in the study if it is experienced with blood testing, ascertained by checking if it provides malaria and/or typhoid blood test services. This was to include PCPs that could adapt to the intervention procedure within the timeframe available for the trial. The inclusion criteria originally stated in the protocol, including the presence of a PCP in the database of the pharmacist council of Nigeria (PCN) and the regular presence of a pharmacist registered with PCN in a PCP, were not assessed because they could not be efficiently evaluated. These changes to the inclusion criteria were made before the first PCP was recruited. In the first instance, an eligibility assessment of PCPs was done by a member of the research team. They presented as a client to PCPs and asked pharmacy staff whether they provide malaria and typhoid test services. They told pharmacy staff that they would return for the test(s) where necessary. Following the assessment, PCPs that met the inclusion criteria were approached by the principal investigator, and relevant details were verbally provided to their managers. If a manager did not provide consent, another eligible PCP was approached. Two written consent forms were given to the managers, who provided consent, to sign. The consent forms were equally signed by the principal investigator, who then left with one copy.

### Randomization and masking

Eligible PCPs were randomly assigned to the intervention and control arms on a 1:1 basis leveraging the stratified block randomization technique. This technique, rather than the block randomization originally stated in the protocol, was used to ensure that PCPs were uniformly distributed between the study arms with respect to the baseline value of the primary outcome. The decision to use this technique was made before the first PCP was recruited. The statistical analysis plan was updated accordingly to reflect this change. The stratification variable was based on the percentage of encounters (visits) in which an antibiotic was dispensed by PCPs at baseline (details of how this information was collected are in the “Outcomes” section). It had three strata: low, moderate, and high. PCPs that dispensed antibiotics in < 70%, 70-89%, and ≥ 90% of visits at baseline were, respectively, categorized into the low (four PCPs), moderate (ten PCPs), and high (six PCPs) strata. Each stratum had a separate list. The random sequence for each stratum (consisting of 1s and 2s) was generated using a research randomizer [12] and coded (specifying which number represented intervention) by an individual who was not part of the trial team. Two sets of random numbers (two numbers per set), five sets of random numbers (two numbers per set), and three sets of random numbers (two numbers per set) were, respectively, generated for the low, moderate, and high strata. By following the random sequence after it was shared with the research team, PCPs within the list of each stratum were assigned 1 or 2 sequentially. The code (specifying which number represented intervention) was shared with the trial team after the assignments had been finalized. PCPs were not masked to treatment assignment. The simulated clients who collected data were masked.

### Interventions

PCPs in the intervention arm were provided with CRP test kits (the kits were specially produced for the trial by Zhuhai Encode Medical Engineering Co., Ltd, China) and other test materials, and their staff were trained on how to use the kits and how to use test results to distinguish viral and bacterial etiologies in patients with suspected RTI without prescriptions in order to make more effective decisions regarding antibiotic dispensation. The CRP test kits were semi-quantitative in nature. The cost of a single CRP test kit was 0.75 $. The test involves using finger prick blood to assess the level of CRP in the blood. Each test takes just over 5 minutes. It has been shown that CRP concentrations >30 mg/l in patients with RTI are of great importance in the identification of RTI that should be managed with antibiotics [13]. Among patients with RTI, this is best interpreted as those with CRP concentrations of >30 mg/l are likely to have bacterial RTI, while those with CRP concentrations of ≤30 mg/l are likely to have viral RTI. Other studies suggest that it is not unusual for many patients with viral RTI to have CRP concentrations >30 mg/l, albeit patients with viral RTI are unlikely to have CRP concentrations >100 mg/l [10,14]. Considering these, regarding managing patients with suspected RTI without prescriptions, pharmacy staff were advised not to dispense antibiotics to patients with CRP concentrations <30 mg/l, to use their clinical judgment to come to a decision when patients have CRP concentrations ≥30 mg/l but <100 mg/l, and to dispense antibiotics when patients have CRP concentrations ≥100 mg/l. They were not discouraged from using their professional judgment where necessary, regardless of CRP test results.

Training, which included practical demonstration, was carried out in each PCP by a pharmacist and a medical laboratory technician. The training was delivered, by the same professionals, in the same way across the board. The staff of PCPs were provided with training materials. They were asked to charge no more than 400 Nigerian Naira (approximately 1 $) per CRP test in order to ensure that low-income patients were not excluded. They were followed up regularly throughout the post-intervention period, and any problems they had were addressed. PCPs in the control arm received no intervention.

### Outcomes

The primary outcome was the rate at which antibiotics were dispensed to patients with RTI without prescriptions, that is, the proportion of visits in which antibiotics were dispensed. The secondary outcome was the rate at which a medical test for identifying bacterial RTI was conducted to inform decisions regarding non-prescription antibiotic dispensation to patients with RTI, that is, the proportion of visits in which relevant tests were conducted.

Data were collected by simulated clients who were students of Nnamdi Azikiwe University, Nigeria, recruited by the research team. The simulated clients were trained on what the data collection entailed. The training was fundamentally delivered twice, shortly after the simulated clients were recruited and a few days before data collection started. Each training consisted of a 2-hour lecture. The simulated clients were provided with training materials. Following the second training, they were engaged in a 6-hour role play in which some members of the research team acted as pharmacy staff while the simulated clients acted as patients with RTI. Any mistakes made by them were corrected by members of the research team. On the day that each simulated client would collect data, the research team member who supervised the data collection discussed essential parts of the data collection protocol with them.

At baseline, 30 simulated clients collected data over 30 data collection days. On each data collection day, just one simulated client visited and collected data from the 20 PCPs under the supervision of a member of the research team who monitored each visit from a distance. Similarly, post-intervention, 30 simulated clients collected data over 30 data collection days. On each data collection day, just one simulated client visited and collected data from the 20 PCPs under the supervision of a member of the research team who monitored each visit from a distance. The PCPs did not anticipate the visits by the simulated clients. Male and female simulated clients mostly collected data on alternate data collection days. On their data collection day, each simulated client was told the symptoms of RTI to complain about. They complained of the same symptoms across all the 20 PCPs. The symptoms complained of over the 30 days of data collection at baseline, and post-intervention can be found in the appendix. These symptoms were developed leveraging the common symptoms of RTI as documented in the literature [15], [16], [17].

After presenting their symptoms without any prescriptions, they asked the pharmacy staff what the best treatments for their symptoms were. They did not request any particular treatments. They did not admit to having any other symptoms outside what they had been told to complain about. They also did not admit to already visiting a doctor or taking any medications. They noted if relevant medical tests were ordered by pharmacy staff to determine suitable treatments and memorized any drugs they recommended. To aid memorization, they mostly memorized the brand names of drugs, these were subsequently categorized as antibiotic or non-antibiotic by a pharmacist. To ensure good simulation, they paid for tests ordered and/or drugs recommended by pharmacy staff. On visits where the budget for drugs was exceeded, they told pharmacy staff that they would return at a later time to purchase recommended drugs. At the end of each visit, they recorded the data collected on a structured data collection form held by the member of the research team who supervised them.

### Statistics

The number of PCPs included in this trial, and the number of visits assessed in each PCP at baseline and post-intervention was based on the recommendations of the World Health Organization [18] regarding the number of facilities and visits to be assessed when evaluating the impact of interventions on drug use practices in healthcare facilities. According to the recommendation, there should be at least ten healthcare facilities in the intervention arm and ten healthcare facilities in the control arm, and data should be collected from at least 30 clinical visits/encounters in each of the healthcare facilities at baseline and post-intervention.

Missing post-intervention data were multiply imputed in R (version 4.2.1) using the jomoImpute function of the mitml package (version 0.4.3) [19]. For the multiple imputation regarding the adjusted analysis, the multiple imputation model included the primary and secondary outcome variables as target variables; treatment status, stratification, and simulated client gender variables as fixed effect predictor variables; and PCP identification variable as a cluster indicator variable. The difference in the multiple imputation regarding the crude analysis was that the fixed effect predictor variable was just the treatment status variable. Regarding missing baseline data, missing primary outcome data were multiply imputed using jomoImpute, with the primary outcome variable as the target variable and the PCP identification variable as the cluster indicator variable. In every case of multiple imputation, 20 imputations were generated because this number of imputations is adequate [20]. Convergence was assessed using the potential scale reduction factor.

Analyses were by intention-to-treat. Relevant frequencies were computed using SPSS Statistics 24. Relevant percentages were then computed accordingly. For the adjusted analysis, using the glmer function of the Ime4 package (version 1.1.30) in R [21], a generalized linear mixed-effects model based on random intercept; with the treatment status, stratification, and simulated client gender variables as fixed effects, and PCP identification variable as a random effect; was used to ascertain if there was a significant difference in the rate at which antibiotics were dispensed in the intervention and control arms after the intervention. The difference in the crude analysis was that the fixed effect was just the treatment status variable. The random effect was used to account for any correlation in the data repeatedly collected from each PCP. The stratification variable was taken into account because not doing so could lead to wrong *P*-values and confidence intervals (CIs) [22]. Analysis was based on the multiply imputed dataset—multiple imputation analysis, and also based on the original dataset with missing data—complete case analysis.

## Results

**Figure 1:**
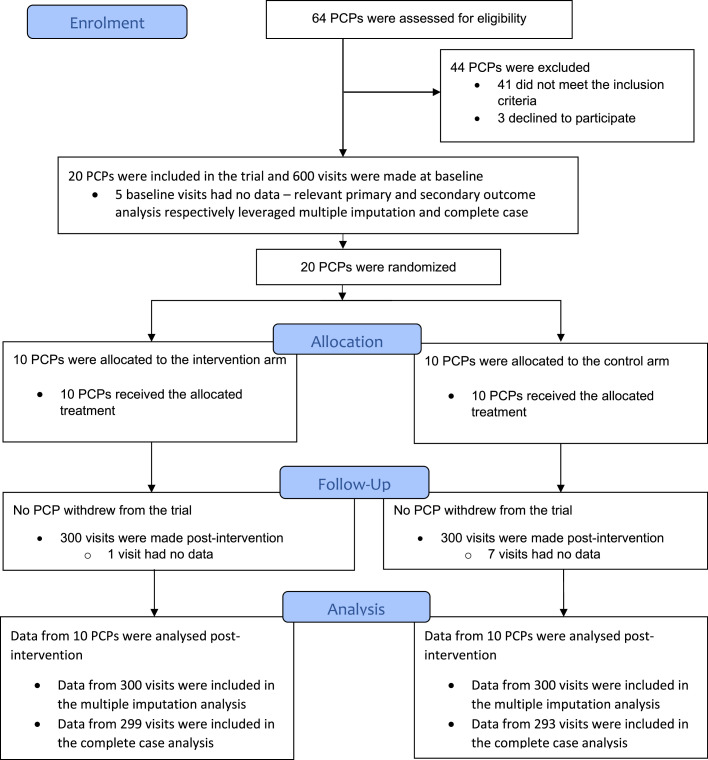
Trial profile. PCP, private community pharmacy.

Sixty-four PCPs were assessed for eligibility. Forty-one did not meet the inclusion criteria. Recruitment of PCPs took place from January 21, 2022, to February 17, 2022. Three PCPs that met the inclusion criteria were excluded because their managers did not provide consent. Twenty PCPs that met the inclusion criteria were included in the trial and randomized. Ten PCPs were randomly assigned to each of the intervention and control arms. They were all treated accordingly. None of the PCPs that were randomized withdrew. The trial ended on July 23, 2022, when post-intervention data collection was completed. In five of the 600 visits (0.83%) at baseline, no data were collected. For the control arm, in seven of the 300 (2.33%) visits post-intervention, no data were collected. For the intervention arm, in one of the 300 visits (0.33%) post-intervention, no data were collected. These were mostly because the concerned PCPs were closed or did not have relevant drugs in stock when simulated clients visited. Please see Figure 1 for more information.

The simulated clients that collected data at baseline and post-intervention mostly had similar demographic characteristics (Table 2). PCPs in the intervention and control arms had similar characteristics with respect to the baseline value of the outcomes (Table 3).

**Table 2:** Demographic characteristics of simulated clients who collected post-intervention and baseline data.

		Post-intervention (n = 30)	Baseline (n = 30)
Gender	Female	15 (50%)	14 (46.67%)
	Male	15 (50%)	16 (53.33%)
Age	18-21	11 (36.67%)	17 (56.67%)
	22-25	19 (63.33%)	13 (43.33%)

Data in the post-intervention and baseline columns are number of simulated clients (%).

**Table 3:** Characteristics of private community pharmacies regarding the percentage of visits in which antibiotics were dispensed, and the percentage of visits in which relevant tests were conducted, at baseline.

	Analysis	Percentage	Relative interpretation	Intervention (n = 10)	Control (n = 10)
Visits in which antibiotics were dispensed	Multiple imputation	< 70%	Low	2 (20%)	2 (20%)
		70-89%	Moderate	5 (50%)	5 (50%)
		≥ 90%	High	3 (30%)	3 (30%)
Visits in which relevant tests were conducted	Complete case	0%	-	10 (100%)	10 (100%)

Data in the intervention and control columns are number of private community pharmacies (%).

Regarding the primary outcome in the adjusted analysis (Table 4), in the multiple imputation analysis, antibiotics were dispensed to simulated clients in 209/300 (69.67%) and 256/300 (85.33%) visits in the intervention and control arms, respectively, (odds ratio = 0.279, 95% CI = 0.107-0.726; *P*-value = 0.0090; absolute reduction in antibiotic dispensation = 15.66%). Regarding the primary outcome in the crude analysis (Table 4), in the multiple imputation analysis, antibiotics were dispensed to simulated clients in 208/300 (69.33%) and 256/300 (85.33%) visits in the intervention and control arms, respectively, (odds ratio = 0.299, 95% CI = 0.098-0.911; *P*-value = 0.034; absolute reduction in antibiotic dispensation = 16%).

**Table 4:** Post-intervention visits in which antibiotics were dispensed.

		Intervention	Control	Odds ratio (95% confidence interval)	*P*-value	AE
Adjusted analysis	Multiple imputation	209/300 (69.67%)	256/300 (85.33%)	0.279 (0.107-0.726)	0.0090	15.66%
	Complete case	208/299 (69.57%)	250/293 (85.32%)	0.273 (0.104- 0.716)	0.0083	15.75%
Crude analysis	Multiple imputation	208/300 (69.33%)	256/300 (85.33%)	0.299 (0.098- 0.911)	0.034	16%
	Complete case	208/299 (69.57%)	250/293 (85.32%)	0.297 (0.097-0.907)	0.033	15.75%

AE, absolute effect.

Data in the intervention and control columns are events/n (%). Data in AE are the absolute difference between the percentages in the intervention and control columns.

Regarding the secondary outcome (Table 5), in the multiple imputation analysis, medical tests for identifying bacterial RTI (CRP tests) were conducted on simulated clients in 65/300 (21.67%) and 0/300 (0%) visits in the intervention and control arms, respectively. Based on the complete case analysis, antibiotics were not subsequently dispensed in 28/64 (43.75%) of the visits in which CRP tests were conducted.

**Table 5:** Post-intervention visits in which relevant tests (C-reactive protein tests) were conducted, with clarification regarding those without subsequent antibiotic dispensation.

	Analysis	Intervention	Control	AE
Visits in which relevant tests were conducted	Multiple imputation	65/300 (21.67%)	0/300 (0%)	21.67%
	Complete case	64/299 (21.40%)	0/293 (0%)	21.40%
Visits without antibiotic dispensation following relevant tests	Complete case	28/64 (43.75%)	-	-

AE, absolute effect.

Data in the intervention and control columns are events/n (%). Data in AE are the absolute difference between the percentages in the intervention and control columns. The multiple imputation model is that of the adjusted analysis.

## Discussion

This is the first study to assess the impact of access to CRP test kits—and staff training on how to use them in RTI management—on the non-prescription dispensation of antibiotics for RTI in PCPs, a situation that is largely seen in many resource-limited settings [9,23,24]. Previous studies that assessed the impact of access to CRP test kits—and staff training on how to use them in RTI management—in PCPs [25,26] focused on how the rate at which patients subsequently visit a general practitioner with the hope of receiving an antibiotic prescription got affected.

This study shows that access to CRP test kits—and staff training on how to use them in RTI management—in PCPs reduces the non-prescription dispensation of antibiotics for RTI, as there was an absolute reduction—in the non-prescription dispensation of antibiotics—of 15.66% and 16%, based on the multiple imputation regarding the adjusted analysis and crude analysis respectively.

Before making treatment decisions, PCPs in the intervention arm conducted CRP tests on simulated clients in 21.40% of visits and 21.67% of visits, based on the complete case analysis and multiple imputation analysis, respectively. No such tests were conducted in the control arm. The results of the CRP tests (that is, viral or bacterial or uncertain) were not explicitly assessed. However, considering that the simulated clients were healthy adults, they were all expected to have CRP levels of <30 mg/l (and hence the CRP tests should suggest that they require no antibiotics). Antibiotics were not dispensed in 43.75% of the visits in which CRP tests were conducted, based on the complete case analysis.

Although the percentage of visits in which CRP tests were conducted is not quite high, the observed uptake of CRP testing suggests that CRP testing in PCPs is feasible. At the end of the trial, key pharmacy staff in the intervention arm were interviewed to ascertain any challenges they had regarding CRP testing. This will be reported separately and will, hopefully, provide insight into strategies that could be used to improve the uptake of CRP testing in future interventions of this kind.

The findings of this trial are similar to that of a trial conducted in Vietnam, a resource-limited setting, to assess the impact of access to CRP test kits—and staff training on how to use them in RTI management—in primary healthcare centers on antibiotic use for RTI. In the trial, Do et al. [11] reported that access to CRP test kits—and staff training on how to use them in RTI management—reduced antibiotic prescribing for RTI by 20%. This suggests that access to CRP test kits—and staff training on how to use them in RTI management—is as effective in reducing healthcare practitioners’ offer of antibiotics for RTI in PCPs as it is in primary healthcare centers in resource-limited settings.

Given that PCPs play an important role in the unnecessary use of antibiotics for RTI in resource-limited settings as they frequently dispense antibiotics to patients with RTI without prescriptions [9,23,24], an intervention of this nature has the potential to improve antibiotic use for RTI and therefore reduce the spread of antibiotic resistance when implemented in such settings. If economic analysis shows that the cost of implementing this intervention in resource-limited settings is manageable, then—all things being equal—its implementation by relevant stakeholders in such settings is recommended, as this would be very helpful in the fight against antibiotic resistance.

Regarding generalisability, antibiotic dispensation for RTI in children was not captured as all the simulated clients were adult university students who were the only population group readily available to work as simulated clients within the resources available for the study. This may limit the generalizability of the findings of this study to the pediatric population. Additionally, it may be that PCPs that fail to meet the inclusion criteria differ significantly—with respect to blood testing experience—from those that were included in the study, thereby threatening the generalizability of the findings of this study to them. However, this is unlikely to be the case. Malaria and typhoid tests were preferentially used to assess blood testing experience solely because the concerned conditions are not sensitive as to make the individual who assessed eligibility uncomfortable. Most PCPs that fail to meet the inclusion criteria are likely to be equally experienced with blood testing by virtue of offering other blood test services, including blood glucose test, which is routinely offered by PCPs in Nigeria [8].

Regarding limitations, this study did not investigate what would happen if patients without prescriptions directly requested to be given antibiotics for their RTI rather than merely complaining about their RTI symptoms. This could be addressed in future trials. In addition, although pharmacy staff did not anticipate the visits by the simulated clients, it is not possible to say with certainty that there was no Hawthorne effect. Therefore, this effect may have contributed to some of the effects observed in this study.

Access to CRP test kits—and staff training on how to use them in RTI management—in PCPs in Nigeria improved the objective assessment of antibiotic needs in patients with RTI without prescriptions and reduced the dispensation of antibiotics to such patients. All things being equal, if economic analysis shows that the cost of implementing this intervention in resource-limited settings is manageable, its implementation by relevant stakeholders in such settings is recommended, as this would help reduce the burden of antibiotic resistance.

## Funding

This trial was funded by the Royal Society of Tropical Medicine and Hygiene, UK, in partnership with the National Institute for Health and Care Research, UK.

## Ethical approval

This trial was approved by the Research Ethics Committee of Chukwuemeka Odumegwu Ojukwu University Teaching Hospital, Awka, Nigeria. Assigned Number: COOUTH/CMAC/ETH.C/VOL.1/FN:04/136.

## Author contributions

AO conceived the idea, designed the trial, and secured funding. AO developed the protocol, obtained ethics approval, and registered the trial. AO and EO implemented the trial. AO processed and analysed the data. AO, OE, and EO had full access to, and verified, the data. AO drafted the manuscript. AO, OE, and EO revised and approved the manuscript for submission. AO had final responsibility for the decision to submit for publication.

### Registration

This trial was prospectively registered at the Pan African Clinical Trials Registry. Its registration number is PACTR202111611256940.

### Trial protocol

The trial protocol will be available to researchers who request it from the corresponding author.

## Declaration of interests

The authors declare no competing interests.

## Appendix Supplementary materials

Image, application 1
