# Supplementary material for: Impact of point-of-care C-reactive protein testing intervention on non-prescription dispensing of antibiotics for respiratory tract infections in private community pharmacies in Nigeria: a cluster randomized controlled trial
Source: Int J Infect Dis. 2023 Feb;127:137–43. doi: 10.1016/j.ijid.2022.12.006 (PMC9876806; doi:10.1016/j.ijid.2022.12.006)
Supplement: Supplementary file 1 [file mmc1.pdf]

Appendix

Supplementary table 1: Symptoms complained of over the 30 days of data collection at baseline and post-intervention

Sore throat that started yesterday, in addition to dry cough since this morning.
Itchy throat, in addition to cough that produces colourless sputum, that started today.
Dry cough and runny nose that started yesterday morning.
Sore throat, in addition to the production of colourless sputum when the throat is cleared, that started yesterday.
Dry cough and sneezing that started today.
Dry cough that started 7 days ago, in addition to sore throat since yesterday.
Cough that produces yellow sputum, in addition to runny nose, that started 3 days ago.
Sore throat that started 2 days ago, in addition to dry cough since yesterday evening.
Itchy throat, in addition to cough that produces yellow sputum, that started this morning.
Dry cough, in addition to sore throat, that started yesterday but got worse this morning.
Sore and itchy throat, in addition to sneezing, that started yesterday morning.
Sore throat, in addition to the production of yellow sputum any time the throat is cleared, that started 2 days ago.
Sore and itchy throat that started yesterday morning.
Cough that produces yellow sputum, in addition to sore throat, that started 2 days ago.
Dry cough and runny nose that started yesterday.
Sore throat that started 2 days ago, in addition to the production of yellow sputum any time the throat is cleared since yesterday.
Dry cough that started 2 days ago, in addition to sore throat since this morning.
Sore throat and stuffy nose that started this morning.
Cough that produces colourless sputum that started 3 days ago, in addition to sore throat since this morning.
Dry cough, in addition to sore throat, that started 4 days ago.
Cough that produces colourless sputum, in addition to stuffy nose, that started 2 days ago.
Sore throat, in addition to the production of yellow sputum any time the throat is cleared, that started 2 days ago.
Sore throat, in addition to cough that produces yellow sputum, that started yesterday evening.
Runny nose and constant sneezing that started 2 days ago, in addition to dry cough since yesterday.
Persistent dry cough, in addition to stuffy nose at night, that started 3 days ago.
Dry cough, in addition to stuffy nose at night, that started 2 days ago.
Dry cough that started 3 days ago.
Runny and stuffy nose, in addition to mild dry cough, that started 2 days ago.
Dry cough and sneezing that started 3 days ago.
Runny nose and dry cough that started yesterday.
